# Chromosome-level assembly of *Triplophysa yarkandensis* genome based on the single molecule real-time sequencing

**DOI:** 10.1038/s41597-023-02900-x

**Published:** 2024-01-05

**Authors:** Jiacheng She, Shengao Chen, Xuan Liu, Bin Huo

**Affiliations:** 1 College of Fisheries, Huazhong Agricultural University, Wuhan, 430070 China; 2 College of Life Sciences and Technology, Tarim University, Alar, 843300 China

**Keywords:** Ichthyology, Comparative genomics, Genome

## Abstract

*Triplophysa yarkandensis*, a species of freshwater fish endemic to Xinjiang, China, is currently classified as endangered. The objective of this study was to obtain the chromosome-level genome of *T. yarkandensis* using PacBio and Hi-C techniques. The PacBio sequencing technology resulted in an assembly of 520.64 Mb, with a contig N50 size of 1.30 Mb. Hi-C data was utilized for chromosome mapping, ultimately yielding 25 chromosome sequences. The success rate of chromosome mapping was 93%, with a scaffold N50 of 19.14 Mb, and a BUSCO evaluation integrity of 94.1%. The genome of *T. yarkandensis* encompasses 25,505 predicted protein-coding genes, with a total of 30,673 proteins predicted. The BUSCO evaluation integrity for predicted protein-coding genes was found to be 91.5%. Additionally, the genome contained a genomic repeat sequence accounting for 27.29% of its total length. Future research employing comparative genomics holds considerable importance in elucidating the molecular mechanisms behind saline-alkali adaptation and ensuring the conservation of biological resources.

## Background & Summary

Food security is a fundamental challenge in the context of human survival and development. Aquatic foods, known as blue foods, are abundant in essential micronutrients and fatty acids while imposing lower environmental burdens. These foods offer protein and valuable nutrients to billions of people, particularly in developing countries^1–4^. In comparison to capture fisheries, aquaculture continues to dominate global blue food production and holds promise for meeting food demand and addressing malnutrition^2,5^. Inland aquaculture, excluding mariculture, significantly contributes to global food security, particularly in the global south^6–8^. However, freshwater resources and arable land pose primary constraints to the growth of inland aquaculture industries. Arid regions, covering approximately 6.1 billion hectares or 41% of the Earth’s land area, constitute a substantial part of the planet’s landmass^9^. Expanding inland aquaculture in arid areas represents a crucial pathway for industry development. Advancements in cultivation techniques have facilitated the robust development of aquaculture in arid regions, particularly in Africa. This not only mitigates food crises to some extent but also drives overall national and societal progress^10^.

The Tarim River, China’s longest inland river, serves as the main river of the southern Xinjiang Autonomous Region^11^. The Tarim River Basin is characterized by arid conditions, including limited precipitation, high evaporation rates, sparse vegetation, minimal runoff, severe water salinization, and a simple native fish fauna^12^. To utilize saline-alkaline water resources and diversify animal protein sources, several euryhaline non-native fish species, such as *Ctenopharyngodon della*, *Cyprinus carpio*, and *Carassius auratus*, have been introduced for saline-alkaline fisheries. Unfortunately, this practice poses significant ecological risks^13–15^. Furthermore, various water storage and diversion projects have been implemented along the upstream to downstream axis of the Tarim River Basin to alleviate water scarcity^16,17^. The combination of hydraulic engineering projects and the introduction of non-native fish species for aquaculture can facilitate fish invasions and lead to a drastic decline in indigenous fish populations and faunal homogeneity^18–20^.

Breeding euryhaline native fish species may offer an effective solution for mitigating the ecological impacts resulting from biological invasions and conserving native fish populations through artificial propagation and release^15,21^. *T. yarkandensis*, exclusively distributed in the Tarim River Basin, is an euryhaline indigenous fish species with aquaculture potential^22–24^. This species is currently under serious threat and has been included in the list of key protected wild animals for the Xinjiang Uygur Autonomous Region (https://www.xinjiang.gov.cn/xinjiang/zfgbml/202301/2dff780e69894c2cbe56a7b7866e58ca.shtml). Elucidating the complete genome of *T. yarkandensis* not only provides insights into breeding techniques but also offers valuable suggestions for its protection. Therefore, this study combines PacBio long-read sequencing and high-throughput chromosome conformation capture (Hi-C) technology to generate a high-quality, chromosome-level reference genome of *T. yarkandensis*. This achievement will assist in developing effective protection strategies for this species and serve as a basis for exploring adaptive evolution in arid regions.

## Methods

### Sample collection and sequencing

**Fig. 1:**
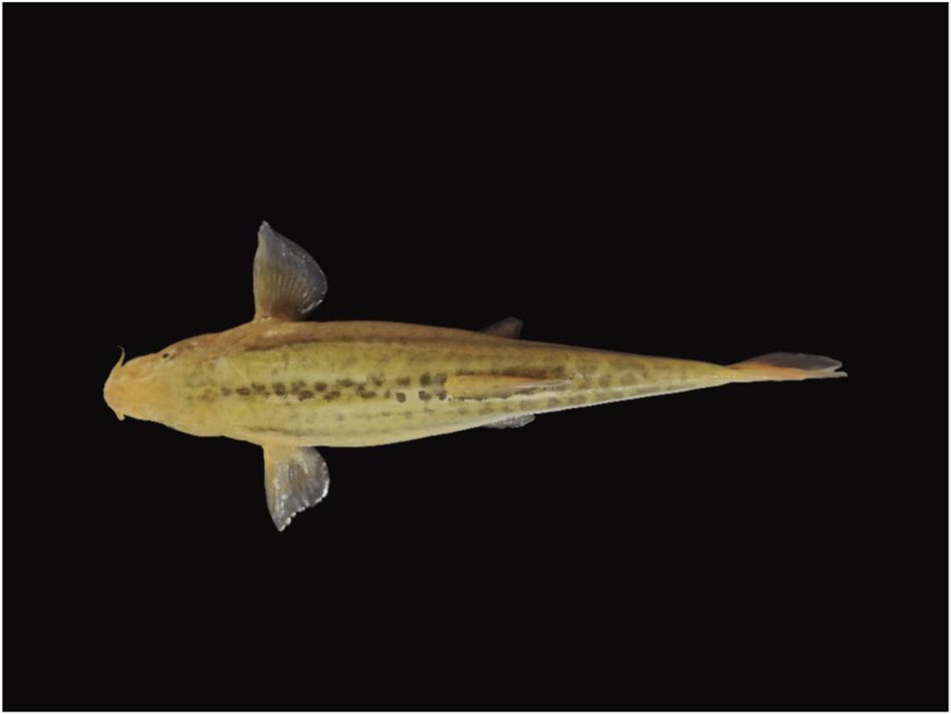
The morphological image of female *Triplophysa yarkandensis* collected in the Yarkant River.

In August 2021, a female *T. yarkandensis* (Fig. 1) was captured at the sampling location of Yarkant River (76°30′56′′ E, 37°59′5′′ N). This species was identified according to their morphological features as described in Fauna Sinica (Osteichthyes: Cyprinifores) and The fishes of the Qinghai-Xizang Plateau. Following anesthesia with MS-222 and disinfection, white muscle tissue was preserved in liquid nitrogen for genomic DNA sequencing. The QIAGEN Genomic-tip 100/G kit was employed for genomic DNA extraction from *T. yarkandensis*, and high-quality DNA was utilized for subsequent library preparation and high-throughput sequencing.

To construct a 20 kb long-read sequencing library (SMRT bell library), 10 µg of *DNA* was utilized. Once the library passed the quality assessment, PacBio Sequel was used for sequencing, following the desired data volume requirements^25^. Sequencing was conducted using the Sequel Binding Kit 2.0, Sequel Sequencing Kit 2.1, and Sequel SMRT Cell 1 M v2, and the resulting data was processed using SMRT LINK 5.0 software. For Hi-C sequencing, the process began with Hi-C biotin labeling and genomic DNA extraction^26^. The captured DNA was subjected to end repair, poly A tailing, adapter ligation, evaluation of PCR amplification cycles, and purification. After qualifying the library inspection, the library was pooled based on the effective concentration and the target offline data volume for HiSeq sequencing.

In order to assist in genomic annotation, total RNA was extracted from six tissues involving kidney, liver, gonad, muscle, brain and gill. The cDNA library was constructed using mixed RNA samples, and the Illumina HiSeq X-Ten platform was employed for sequencing.

### Genome assembly

The SMRTbell libraries were subjected to sequencing on a PacBio Sequel II system. The consensus reads, also known as HiFi reads, were generated using the ccs software (https://github.com/pacificbiosciences/unanimity) with the parameter ‘-minPasses 3’. To enhance the quality and validate the assemblies, we generated 24.6 Gb of PacBio HiFi reads for this specific sample (Table 1). These HiFi reads, which are long (approximately 15 kb) and highly accurate ( > 99%), were assembled using Hifiasm^27^ (https://github.com/chhylp123/hifiasm). To rectify any errors in the primary assembly, Illumina-derived short reads were employed, and remaining errors were corrected using pilon^28,29^ (v1.23). As a result, the *T. yarkandensis* genome assembly reached a total length of approximately 520.6 Mb, consisting of 1707 contigs, with a ContigN50 value of 1.3 Mb (Table 2).

**Table 1:** Sequencing data used for the genome *T. yarkandensis* assembly.

Index	Hic data	PacBio
Library size	350	20000
Raw Reads	877141302	33282948
Raw Base (Gb)	131.6	413.6
Clean Reads	870124172	1685236
Clean Base (Gb)	130.5	24.6
Reads Length	150	14617
Coverage Depth	250.33X	47.24X

**Table 2:** Assembly and annotation statistics of the *T. yarkandensis* genome.

Seq ID	Chr	Contig	Scaffold
Seq number	25	1707	245
Total Base	484949844	520635847	521366357
N50	19284665	1296405	19144862
L50	10	101	11
N90	15406973	115223	14193203
L90	21	702	23
mean	19397993	305000	2128025
median	19144862	85958	54203
max	31126613	6737440	31126613
min	13572666	14703	14703
GC content (%)	37.44	37.68	37.63

**Fig. 2:**
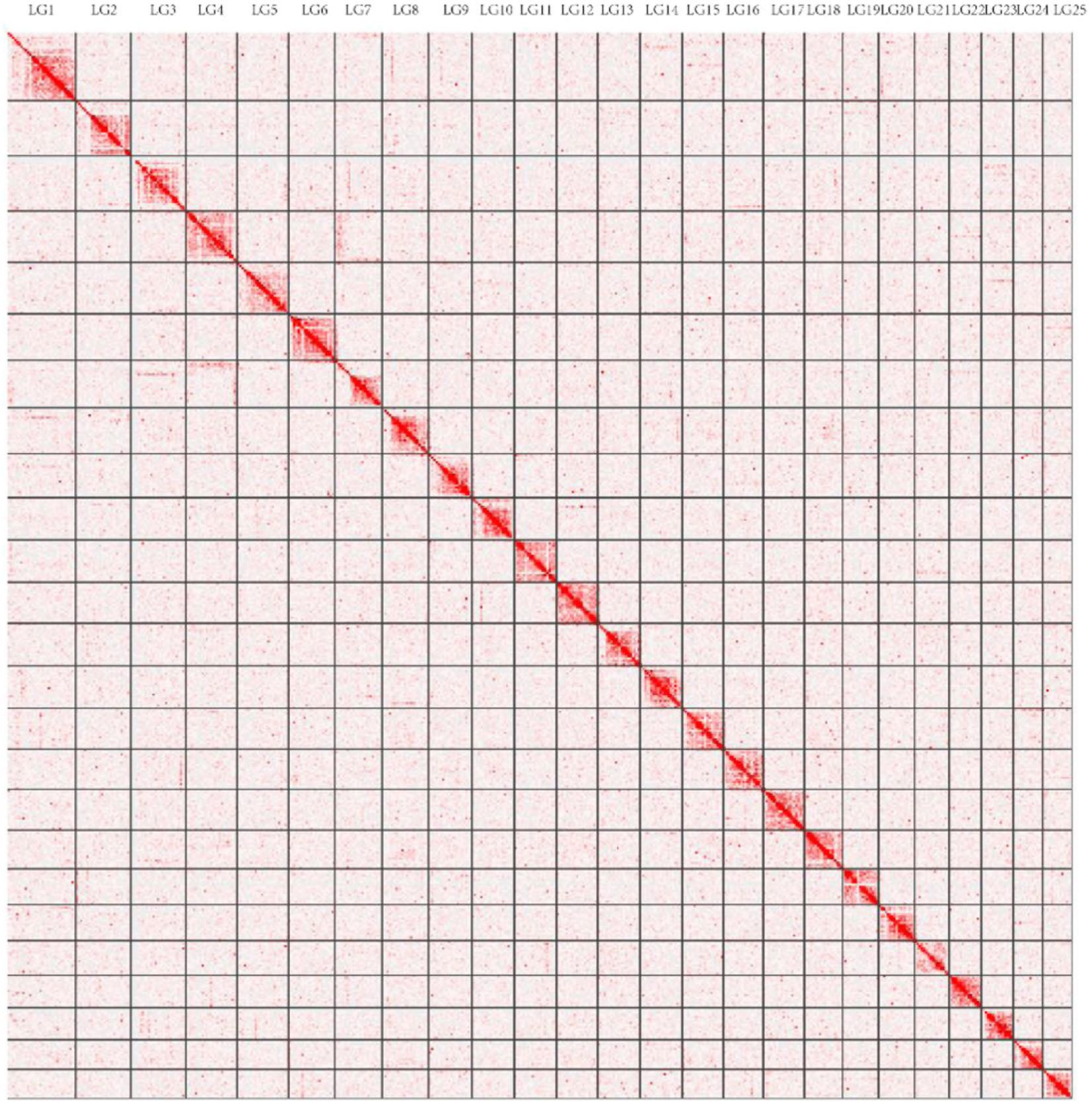
*Triplophysa yarkandensis* genome contig contact matrix using Hi-C data. LG1–25 represented for the 25 pseudo-chromosomes. The depth of red color shows the contact density.

For the anchored contigs, a total of 130.5 Gb of clean read pairs was generated from the Hi-C library (Table 1). These reads were mapped to the polished *T. yarkandensis* genome using BWA (bwa-0.7.17) with default parameters. Paired reads in which the mate was mapped to a different contig were utilized for Hi-C-associated scaffolding. Various types of invalid reads, including self-ligation, non-ligation, Start NearRsite, PCR amplification, random break, Large Small Fragments, and Extreme Fragments, were filtered out using Hicup software. Subsequently, we successfully clustered 1354 contigs into 25 groups using the agglomerative hierarchical clustering method in 3d-DNA (Fig. 2). 3d-DNA was further employed to order and orient the clustered contigs^30–33^. Finally, we obtained the first high-quality assembly at the chromosomal level, with chromosomal lengths ranging from 13.6 Mb to 31.1 Mb, the *T. yarkandensis* genome was obtained with 245 scafolds and a total length of 521,366,357 bp, encompassing 93% of the total sequence (Table 2).

### Repetitive sequence annotation

In our study, we employed a combination of two methods, namely homology-based and de novo prediction, to identify repeat contents in the *T. yarkandensis* genome. For the homology-based analysis, we used RepeatMasker (open-4.0.9) with the Repbase TE library to identify known TEs within the genome. In addition, we employed RepeatModeler (http://www.repeatmasker.org) for de novo prediction. RepeatModeler integrates two core de novo repeat-finding programs, RECON (v1.08) and RepeatScout (v1.0.5), to comprehensively discover, refine, and classify consensus models of potential interspersed repeats in the *T. yarkandensis* genome^34,35^. Moreover, we conducted a de novo search specifically for long terminal repeat (LTR) retrotransposons using LTR FINDER (v1.0.7), LTR harvest (v1.5.11), and LTR_retriever (v2.7) against the *T. yarkandensis* genome sequences^36,37^. Tandem repeats were identified using the Tandem Repeat Finder^38^ (TRF) package, and Simple Sequence Repeats (SSRs) were detected using MISA (v1.0). Finally, we merged the library files generated from both methods and utilized RepeatModeler to determine the repeat contents. Based on these analyses, we identified a total of 149.99 Mb of repeats in the *T. yarkandensis* genome (Table 3).

**Table 3:** Repeat sequence results statistics of *Triplophysa yarkandensis* genome.

Repeat type	Total size
DNA/hAT-Ac	480371
DNA/hAT-Charlie	333228
DNA/hAT-Tip100	177572
DNA/Kolobok-T2	545711
DNA/Maverick	61437
DNA/PIF-Harbinger	590381
DNA/PIF-ISL2EU	173990
DNA/PiggyBac	78885
DNA/TcMar	18625
DNA/TcMar-ISRm11	139673
DNA/TcMar-Tc1	3259895
LINE/L1	950421
LINE/L1-Tx1	262167
LINE/L2	8721801
LINE/R2-Hero	108755
LINE/Rex-Babar	4215861
LINE/RTE-X	86102
Low complexity	1877532
LTR/DIRS	7204242
LTR/ERV1	395791
LTR/Gypsy	7205801
LTR/Pao	94606
LTR/unknown	1449764
Simple repeat	23569351
Unknown	87983554
Total	149985516

### Non-coding RNA annotation

To identify specific gene categories in the *T. yarkandensis* genome, we utilized various algorithms and databases. The tRNAscan-SE (v1.3.1) algorithm with default parameters was employed to detect tRNA genes. tRNA molecules act as adaptors in biological processes, bridging the three-letter genetic code in messenger RNA (mRNA) with the twenty-letter amino acid code in proteins. For the identification of rRNA genes, we used RNAmmer (v1.2) with the parameters “-S euk -m lsu,ssu,tsu”. rRNAs are integral components of the ribosome and play a crucial role in protein synthesis. snoRNAs, a class of small RNA molecules, guide chemical modifications of other RNAs, including ribosomal RNAs, transfer RNAs, and small nuclear RNAs. MiRNAs and snRNAs were identified by CMSAN (v1.1.2) software against the Rfam (v14.0) database with default parameters (Table 4)^39–41^.

**Table 4:** The number of the annotated non-coding RNA in the *Triplophysa yarkandensis*.

Type		Number	Total length	Average length	Genome ratio
rRNA	18s_rRNA	2	3615	1807	
	28s_rRNA	2	8398	4199	
	5.8S_rRNA	1	153	153	
	5S_rRNA	1317	154514	117	
tRNA		8946	667801	74	0.0013
snRNA		256	36295	141.78	
snoRNA		18	2312	128.44	

### Protein-coding gene prediction and annotation

To predict protein-coding genes in the *T. yarkandensis* genome, we employed three methods: ab initio gene prediction, homology-based gene prediction, and RNA-Seq-guided gene prediction. Prior to gene prediction, the assembled genome underwent hard and soft masking using RepeatMasker. Ab initio gene prediction was performed using Augustus (v. 3.3.3)^42^. The gene predictors’ models were trained using a set of high-quality proteins derived from the RNA-Seq dataset. For homology-based gene prediction, we utilized MAKER (v. 2.31.10)^43^. Protein and transcript sequences were aligned to the genome assembly, and coding genes were predicted using maker with default parameters. RNA-Seq-guided gene prediction involved aligning clean RNA-Seq reads to the genome using hisat2 (v2.0.0). Gene structures were generated using Trinity (v2.3.2), Transdecoder (v2.01), and MAKER. To integrate the predictions from the three methods and generate gene models, we employed EVidenceModeler (EVM, v1.1.1)^44^. The resulting output comprised consistent and non-overlapping sequence assemblies, which described the gene structures. Overall, a total of 25,505 protein-coding genes with an average length of 158,469 bp were predicted in the assembled *T. yarkandensis* genome. The predicted protein-coding gene BUSCO integrity using the *Actinopterygii* odb9 database was 91.5%. The number of predicted proteins was 30,673.

For inferring gene functions, we conducted alignments to various protein databases, including the National Center for Biotechnology Information (NCBI) Non-Redundant (NR), TrEMBL, KOG, and SwissProt, using BLASTP (NCBI BLAST v2.6.0+). Additionally, we utilized the Kyoto Encyclopedia of Genes and Genomes (KEGG) database with an E-value threshold of 1E-5. Protein domains were annotated using PfamScan (v1.6) based on the PFAM and InterPro protein databases. Gene Ontology (GO) IDs for each gene were obtained from Blast2GO. In total, approximately 23,288 (about 91%) of the predicted protein-coding genes in *T. yarkandensis* could be functionally annotated with known genes, conserved domains, and Gene Ontology terms (Table 5).

**Table 5:** Protein-coding gene prediction for *T. yarkandensis* genome.

Database	Annotated number	Annotated ratio
GO	13165	51%
KEGG	12049	47%
KOG	15154	59%
NR	23145	90%
Pfam	19806	77%
SwissProt	19971	78%
TrEMBL	22738	89%
Total	23288	91%

## Data Records

All raw data of the whole genome have been deposited into the National Center for Biotechnology Information (NCBI) SRA database under BioProject accession number PRJNA995909 The genomic PacBio sequencing data were deposited in the SRA at NCBI SRR25357712^45^ and the Hi-C sequencing data were deposited in the SRA at NCBI SRR25343507^46^. The RNA sequencing data were deposited in the SRA at SRR26377503^47^. The assembled genome was deposited in the NCBI Genome with the accession number GCA_033220385.1^48^. Genome annotations, along with predicted coding sequences and protein sequences, can be accessed through the Figshare^49^.

## Technical Validation

### Evaluation of the quality of genomic DNA and RNA

Before constructing the DNA library, we assessed the purity (OD260/280 and OD260/230) and concentration of the genomic DNA using Nanodrop (LabTech, USA). To precisely measure the concentration of genomic DNA, we employed Qubit (Thermo Fisher Scientific, USA). By comparing the Qubit concentration with the Nanodrop concentration, we determined the sample purity. Additionally, the integrity of the DNA was assessed through agarose gel electrophoresis (1%). RNA purity and integrity were determined using NanoPhotometer spectrophotometer (Implen, USA) and Agilent 2100 bioanalyzer (Agilent Technologies, USA).

### Genome assembly integrity assessment

**Fig. 3:**
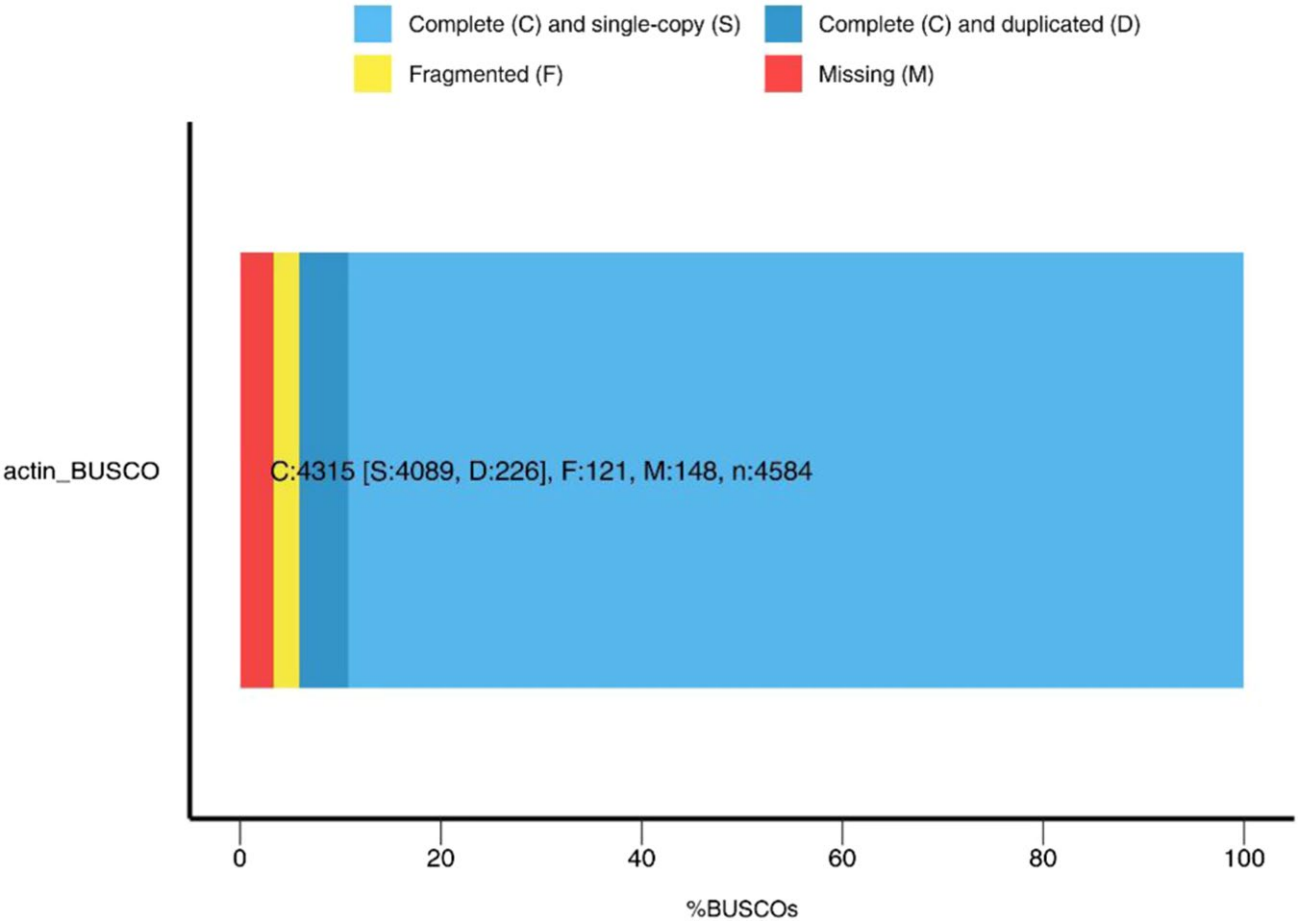
BUSCO assessment results of *Triplophysa yarkandensis* genome. C represents complete BUSCOs, S represents complete and single-copy BUSCOs, D represents complete and duplicated BUSCOs, F represents fragmented BUSCOs, M represents missing BUSCOs, n represents total BUSCO groups searched.

The assembled genome was subjected to BUSCO (v3.1) analysis using OrthoDB to assess its completeness^50^. Overall, the assembled genome identified 94.1% completeness of the BUSCOs (*Actinopterygii* odb9) (Fig. 3).

## Data Availability

No custom scripts or codes were used in the management and verification of the data sets in this study. All software and pipelines used for data processing were executed according to the manuals and protocols of the bioinformatics software cited above. The specific parameters were described if the default parameters were not applied for data analysis.
